# Influence of Geometry and Surrounding Conditions on Fluid Flow in Paper-Based Devices

**DOI:** 10.3390/mi7050073

**Published:** 2016-04-25

**Authors:** Noosheen Walji, Brendan D. MacDonald

**Affiliations:** Faculty of Engineering and Applied Science, University of Ontario Institute of Technology, 2000 Simcoe Street North, Oshawa, ON L1H 7K4, Canada

**Keywords:** paper-based devices, paper microfluidics, imbibition, environmental monitoring

## Abstract

Fluid flow behaviour in paper is of increasing interest due to the advantages and expanding use of microfluidic paper-based analytical devices (known as µPADs). Applications are expanding from those which often have low sample fluid volumes, such as diagnostic testing, to those with an abundance of sample fluid, such as water quality testing. The rapid development of enhanced features in μPADs, along with a need for increased sensitivity and specificity in the embedded chemistry requires understanding the passively-driven fluid motion in paper to enable precise control and consistency of the devices. It is particularly important to understand the influence of parameters associated with larger fluid volumes and to quantify their impact. Here, we experimentally investigate the impacts of several properties during imbibition in paper, including geometry (larger width and length) and the surrounding conditions (humidity and temperature) using abundant fluid reservoirs. Fluid flow velocity in paper was found to vary with temperature and width, but not with length of the paper strip and humidity for the conditions we tested. We observed substantial post-wetting flow for paper strips in contact with a large fluid reservoir.

## 1. Introduction

Microfluidic paper-based analytical devices (µPADs) are a versatile technology capable of facilitating a variety of complex detection and diagnostic processes [1,2,3]. Microfluidics has traditionally involved small fluid volumes (10^−9^ to 10^−18^ L) [2] and µPADs have also been used in applications with small fluid volumes. Processing and analyzing small fluid volumes is critical in many fields, particularly in diagnostics, due to the availability of low fluid volumes and convenience associated with collecting small volumes of fluid, such as finger prick collection of blood samples. µPADs have been developed for a number of diagnostic analytical tests including infectious diseases [4,5,6], and cancer detection and investigation [7]. µPADs can also be used for testing where larger fluid volumes are available as evidenced by the widespread application of µPADs for pregnancy testing using urine samples, and recently there has been a trend towards using µPADs in applications where an abundance of fluid is available, such as environmental testing, specifically for water quality testing. A number of µPADs have been developed for environmental testing including those testing for heavy metals in water sources [8,9,10,11], heavy metals in industrial waste [12], bacterial contaminants [13], airborne contaminants [14,15], and other biological and pollution targets [16,17,18,19,20]. The availability of larger fluid volumes makes it possible to process more fluid through the devices, for example in cases where greater concentration may be desired; thus, the µPADs can be designed with larger sizes and fed from large reservoirs, which can influence the fluid flow behaviour during wicking. To design µPADs capable of exploiting larger fluid volumes we require an understanding of how the parameters associated with larger fluid volumes, such as larger geometry and fluid reservoirs, influence the fluid flow behaviour. We address this need with a detailed experimental study to characterize the fluid flow behaviour in paper with larger dimensions (beyond 5 mm) and fed from large reservoirs.

µPADs are ideal for applications in low-resource settings due to their low cost, ease of use, and passively-driven flow that allows for independence from auxiliary equipment [1,21]. A vital consideration in the successful application of µPADs on a global scale is the various climate zones they are applied in. For example, one area of interest for microfluidic testing is diagnosing HIV and syphilis in Rwanda, with expanding interest from healthcare workers in India and Tanzania [22]. Rwanda has a tropical highland climate with temperatures ranging from 15 to 30 °C and a humidity range of 38% to 100% [23]. A paper-based hepatitis B detection test was encased in a pen format for safe sample containment, with clinical application in Vietnam [4]. Vietnam experiences tropical climates with temperatures between 20 and 35 °C and humidity between 50% and 98% [23]. We are interested in testing for contaminants, specifically arsenic levels, in drinking water sources in tube wells in Bangladesh. The climate zone in Bangladesh is described as a tropical savannah, with temperatures varying from 15 to 40 °C, and humidity levels from 25% to 95% [23]. Fluid flowing in paper-based devices can undergo evaporation during wicking and some µPADs are encased in plastic to prevent this evaporation [4,24,25]. This is particularly important for applications with low fluid volume so as not to deplete the fluid and prevent the strip from drying out before the test is complete. For devices where larger fluid volumes are available it is possible that the fluid flow behaviour will be influenced by evaporation, and the devices may be more susceptible to the evaporation due to their larger sizes and increased exposure to the surrounding conditions. In µPADs where the consistency of results in each test is necessary regardless of the point-of-interest location, a detailed understanding of the influence of temperature and humidity on fluid flow behaviour during wicking is beneficial to the design process.

The analytical capacity of µPADs has been increasing rapidly due to the development of enhanced features such as multi-dimensional device designs [17,26,27,28,29], enrichment-based techniques [30], filtering and multi-step reactions [31], dissolvable fluidic time delays [32], flow control mechanisms such as pumps [33], and analyte concentration and transport after wetting [34]. These enhanced features have enabled more complex chemistries to be incorporated into paper-based platforms, which have expanded the contaminants that can be detected. Many of these contaminants occur in the presence of other similar constituents, and have low detection limits, thus increasing the need for sensitivity and specificity. Engineering highly sensitive and specific µPADs requires precise fluid flow control that enables pre-programming of the chemical reactions to yield predictable and consistent signal readouts. The current demands for precise flow control have necessitated an in-depth understanding of which parameters influence the flow behaviour and what their impact is; therefore, it is particularly important to analyze how the parameters associated with larger fluid volumes and environmental conditions influence the flow behaviour to ensure precise test results.

In this paper, we characterize fluid flow in paper-based devices using an experimental analysis to determine which parameters influence imbibition and need to be considered when predicting flow behaviour in µPADs when there is an abundance of sample fluid volume available. Specifically, we perform experiments to investigate the influence of surrounding temperature and humidity levels, paper machine direction, strip length, and strip width on the wicking behaviour of fluid in a paper strip when the source of the fluid is a large reservoir. This analysis of influential parameters can be incorporated in design considerations for paper-based devices when larger fluid volumes are available for a range of climate conditions.

## 2. Materials and Methods

### 2.1. Preparation of the Paper Strips

We used pure cellulose chromatography paper (Whatman Grade 1 CHR and 17 CHR, GE Healthcare, Mississauga, ON, Canada) for the paper strips. Table 1 gives the properties of these papers, as provided by the supplier. Strip dimensions were printed onto the paper using an inkjet printer (HP Deskjet 2540, Hewlett-Packard, Mississauga, ON, Canada), and the strips were cut using a craft paper cutter.

### 2.2. Experimental Apparatus

**Figure 1:**
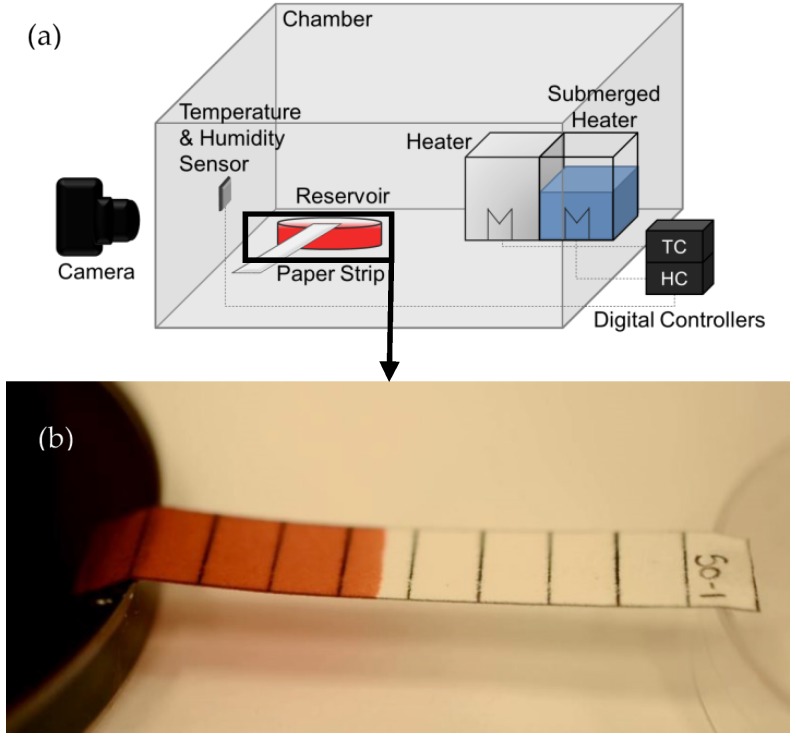
(**a**) Schematic of experimental setup to examine wicking behaviour in µPADs using paper strips dipped in a reservoir; (**b**) paper strip wicking from reservoir during experimentation.

Experiments were conducted in a temperature and humidity controlled chamber measuring 51.5 cm × 41.5 cm × 41 cm, as shown in Figure 1a. The temperature in the chamber was adjusted using a 250 W heat lamp, measured using a digital thermometer, and controlled using a digital temperature controller (TC). The humidity in the chamber was established using a submerged heater in an open container of water, monitored using a digital hygrometer, and controlled using a digital humidity controller (HC). The heater and submerged heater were placed on the right side of the chamber, while the reservoir and sensors were placed on the left side of the chamber, approximately 25 cm away from the heaters. The reservoir was placed 3 cm away from the left wall of the chamber, and the sensors were mounted on the left wall of the chamber, no higher than 10 cm above the reservoir and paper strip, to ensure that the temperature and humidity readings monitored the reservoir and paper strip conditions as accurately as possible. There was no active mixing of the air in the chamber in order to avoid convection, and after changes in conditions the chamber was allowed to reach equilibrium prior to conducting experiments.

The paper strip (Whatman, GE Healthcare, Mississauga, ON, Canada) was folded and placed in a 10 cm diameter petri dish filled with a 12.5 mmol aqueous solution of Allura red food colour dye (Sigma Aldrich, Oakville, ON, Canada) and to keep the strip level the opposite end was supported by an inverted petri dish as shown in Figure 1b. The wicking process was recorded using a Nikon Digital SLR camera (Nikon, Mississauga, ON, Canada) with an AF-S DX Micro-NIKKOR 40 mm f/2.8 G lens (Nikon, Mississauga, ON, Canada). A JEOL 6400 scanning electron microscope (SEM) (JEOL USA, St. Hubert, QC, Canada) was used to take micrograph images.

## 3. Results and Discussion

Experiments were conducted to study the influence of the surrounding temperature and humidity levels, the paper machine direction, and the dimensions of the paper strips including strip length and strip width on the wicking behaviour of fluid in a paper strip fed from a large fluid reservoir.

### 3.1. Influence of Temperature on Wicking

**Figure 2:**
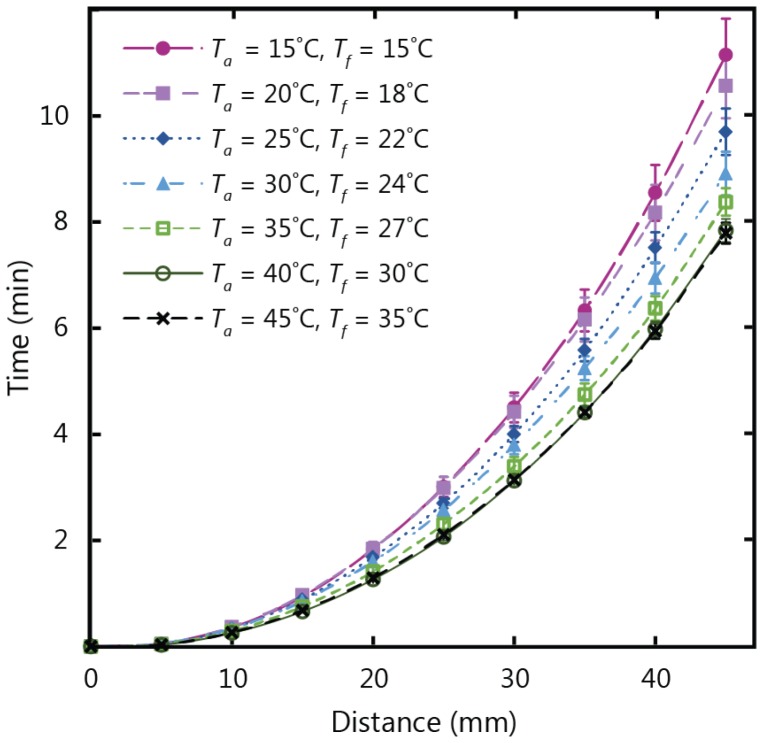
Experimental results for wicking in 1 CHR strips 10 mm in width and 45 mm in length at ambient (*T_a_*) and fluid (*T_f_*) temperature conditions varying from 15 to 45 °C.

**Figure 3:**
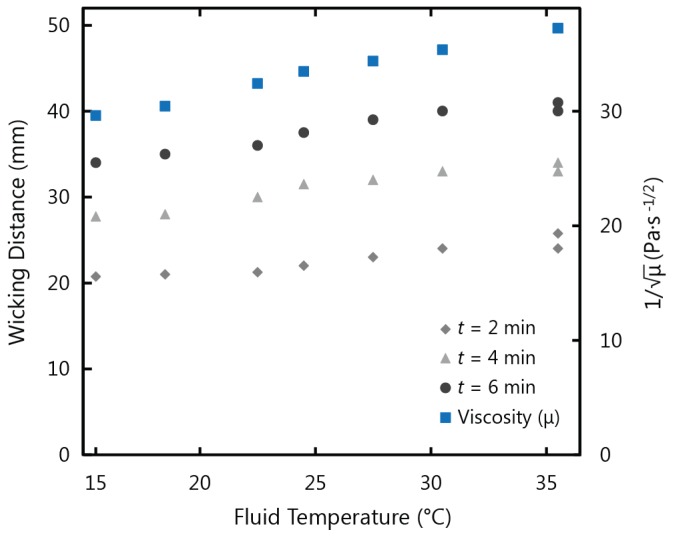
Comparison of the inverse root of viscosity (from the Washburn equation) to experimental data for wicking distance at 2, 4, and 6 min in 1 CHR, 10 mm width strips at varying temperature conditions.

Figure 2 shows the experimental results for fluid flow in paper strips with a 10 mm width and a 45 mm length, tested in temperature conditions ranging from 15 to 45 °C at a fixed relative humidity of 30%. After analyzing the data from the experiments, it was clear that the temperature of the fluid in the reservoir (*T_f_*) was what influenced the wicking behaviour rather than the air temperature (*T_a_*). It can be observed in Figure 2 that the flow profiles follow the typical Washburn behaviour and the distance travelled by the fluid front is proportional to the square root of time [35]:

(1) $$x=\sqrt{\frac{\gamma }{\mu }\frac{\cos\theta }{2}r_{c}t}$$

where x is distance, γ is the surface tension, θ is the contact angle, μ is viscosity of the fluid, $r_{c}$ is the average pore radius, and t is the time. It can also be observed that the total wicking time decreased as temperature increased. In conditions of 15 °C fluid and air temperature, a wicking time of approximately 11 min was required for the fluid front to travel a distance of 45 mm. With a fluid temperature of 35 °C (air temperature of 45 °C), wicking time was reduced by three minutes, and a total duration of approximately 8 min was required for the fluid front to travel 45 mm in the paper strip. These results demonstrate that the time required for the fluid front to travel the length of the paper strip decreases as temperature increases, and correspondingly speed increases. This increase in wicking speed can be attributed to the decrease in the viscosity of water as its temperature increases. To analyze this justification, Figure 3 shows a comparison of the experimental results for wicking distance at 2, 4, and 6 min *versus* the inverse root of viscosity at varying fluid temperatures. This correlation between wicking distance and viscosity follows the Washburn model Equation (1), where the wicking distance is inversely proportional to the root of viscosity. The corresponding dependence of wicking distance and the viscosity term on temperature shown in Figure 3 confirms that the fluid viscosity accounts for the observed variations with temperature.

### 3.2. Influence of Humidity on Wicking

**Figure 4:**
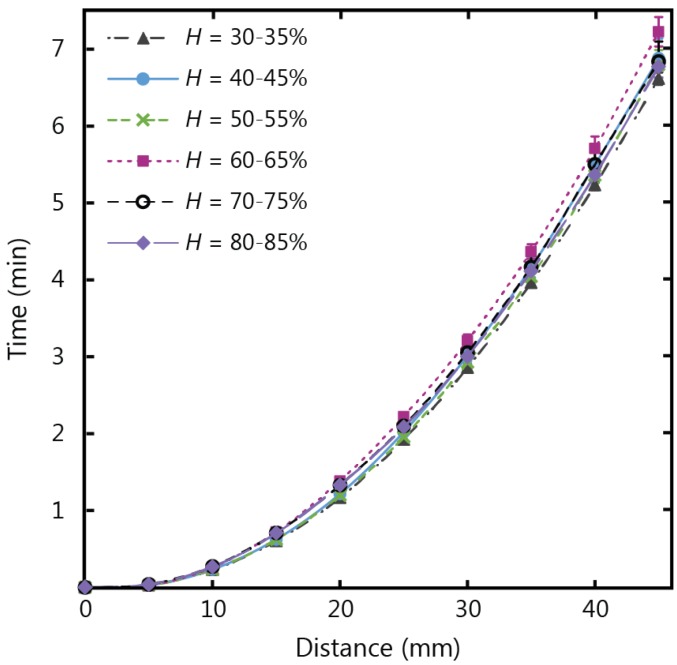
Experimental results for wicking in 1 CHR strips 10 mm in width and 45 mm in length, for humidity (*H*) conditions varying from 30% to 85%.

Wicking in paper strips with the same dimensions as the temperature tests (10 mm width and 45 mm length) were tested in humidity conditions ranging from 30% to 85% relative humidity (*H*), at a fixed air temperature of 20 °C, and the experimental results are shown in Figure 4. Despite the variations in humidity, the wicking time for the fluid to fill the full length of the paper strip had a consistent value of approximately 7 min. There was no observable increase or decrease in wicking velocity beyond the experimental error that corresponded to increases in humidity for the conditions of our experiment. This result indicates that µPADs developed for applications fed from large fluid reservoirs and settings with varying humidity levels can yield consistent flow behaviour regardless of fluctuating levels of humidity.

### 3.3. Influence of Machine Direction on Wicking

During the paper production process the cellulose pulp is laid into sheets, and it has been observed that paper fibres tend to align parallel to the direction of the machine [36,37]. The machine direction of the chromatography paper was labelled by the manufacturer, so an investigation to determine any potential impacts of fibre arrangement could be conducted. Hypotheses for the specific fluid transport mechanism in paper include wicking through capillary-like structures formed by an alignment of pores, or wicking along adjacent fibres [38]. It is expected that fluid flowing in the direction of the fibres will encounter fewer obstructions from the fibres and therefore travel through the paper strip in a shorter time.

**Figure 5:**
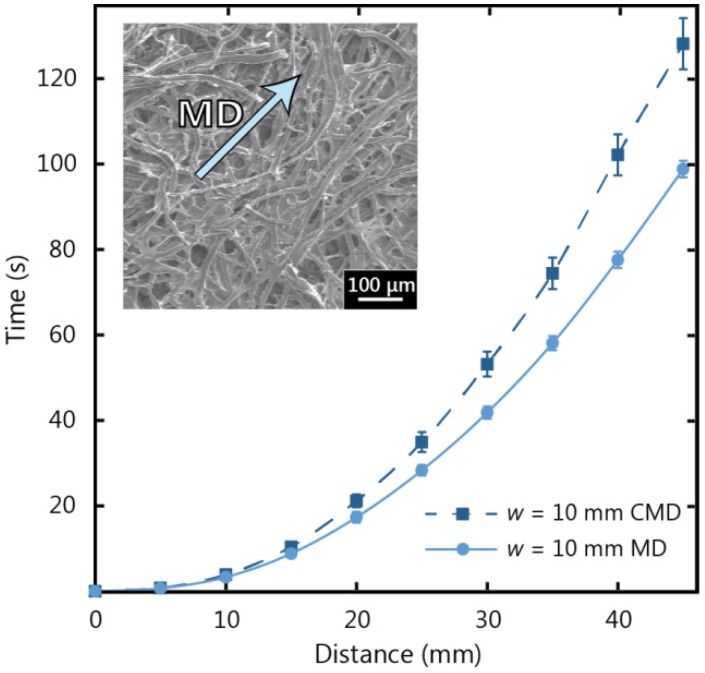
Experimental observations for wicking in a 10 mm wide 17 CHR paper strip, in the machine direction (MD) and cross machine direction (CMD).

The machine direction is labelled in the SEM micrograph inset of Figure 5 for Whatman 17 CHR paper, and the tendency for fibre alignment parallel to machine direction can be observed. Figure 5 shows the results of a comparison for fluid flow in the machine direction and cross direction for Whatman 17 CHR paper with a width (*w*) of 10 mm. It can be seen that fluid flow in the machine direction is faster than in the cross direction and this observation becomes more distinct as wicking distance increases. In Figure 5, it can be seen that the fluid in the machine direction strip wicked to a length of 45 mm 30% faster than in the cross direction strip. For our experimental investigations we chose to use the machine direction, since the chromatography paper is designed to operate in the machine direction and the fluid flow is less inhibited.

### 3.4. Wicking in Paper Strips of Varying Lengths

**Figure 6:**
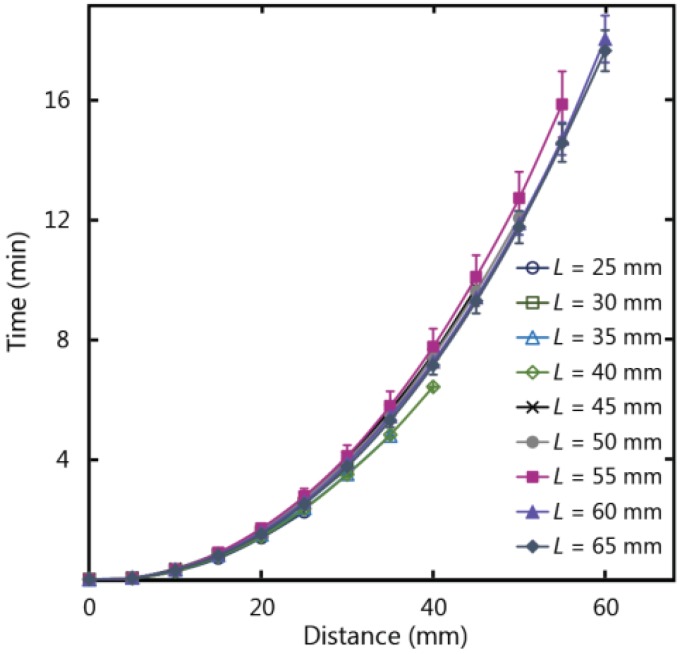
Experimental results for wicking in 1 CHR strips of lengths (*L*) varying from 25 to 65 mm, and a width of 10 mm.

Paper-based devices can be used for analytical testing in situations where there is an abundance of sample fluid volume, for example in water quality testing, and longer paper strip lengths can be exploited for a number of functions including additional reaction steps or to increase the concentration of an analyte by flowing more fluid over a reaction zone. To examine the impacts of paper strip dimensions on fluid wicking, strips of varying lengths (*L*) were tested to ascertain if fluid flow behaviour in paper is influenced by the length of the strip. Figure 6 shows the experimental results for fluid flow in paper strips of varying length and a fixed width of 10 mm, with the fluid temperature at 20 °C (ambient temperature of 22 °C), 25% humidity, and fed from a large fluid reservoir. Experimental data demonstrates that as the lengths of the paper strips increased from 25 to 65 mm, there was no subsequent increase or decrease in flow velocity beyond the experimental error. As such, length is a flexible design parameter for µPADs, and can be adjusted to meet larger size requirements without impacting the wicking behaviour in a paper-based device.

### 3.5. Wicking in Paper Strips of Varying Widths

In situations where paper-based tests are required and there is an abundance of fluid volume, wider paper strip lengths can be exploited for a number of functions including larger test signal output for ease of reading, easier handling of the test by the user, or to increase the amount of fluid collected in a given time. Impacts of the paper strip width were investigated for straight paper strips with a constant width and fed from a large fluid reservoir. In order to examine the influence of the width of the paper strip on fluid flow behaviour, we used paper strips with a length of 45 mm and a range of widths from 5 to 40 mm in increments of 5 mm.

Paper-based devices have been designed and analyzed with width values that fluctuate along the paper strip as a method to control fluid flow by altering the area exposed to the imbibition front and thus slowing down the wicking of the fluid (with increased width/area) or speeding up the flowing fluid (with decreased width/area) [24,26]. It is expected that no changes will occur in the wicking behaviour of the fluid when the width is varied for straight cut strips with a constant width value, since the area is continuous. Previous studies have found that different strip widths influence the flow in straight paper strips with hydrophobic barriers resulting in varying wicking times [39,40]. Songok *et al.* investigated small channel widths of 0.5, 1.0 and 1.5 mm, and found the variation in wicking speed to be due to the shape of the droplet that was feeding the channel from above [39]. Hong and Kim investigated small channel widths of 1, 2, and 4 mm and concluded that the effects of hydrophobic channel boundaries are significant with a width on the order of 1 mm [40]. The slower flow in channels with smaller widths was attributed to the surface tension forces at the hydrophobic boundaries that oppose the flow, and therefore they expect no variation for paper strips with cut edges. Paper strips with cut edges and widths larger than a few millimeters have not been analyzed to determine the influence of width on the fluid flow behaviour and we provide our observations here for straight cut strip widths from 5 to 40 mm.

**Figure 7:**
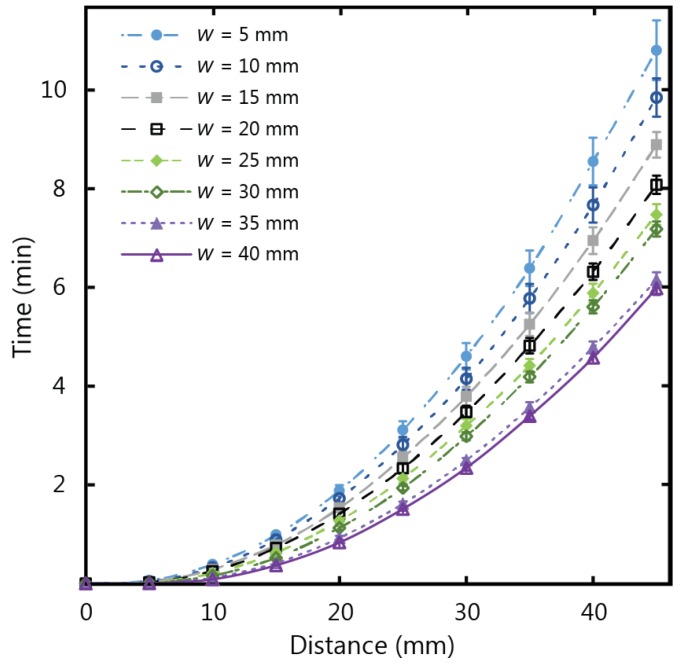
Experimental results for wicking in 1 CHR strips of widths (*w*) varying from 5 to 40 mm, and a length of 45 mm.

**Figure 8:**
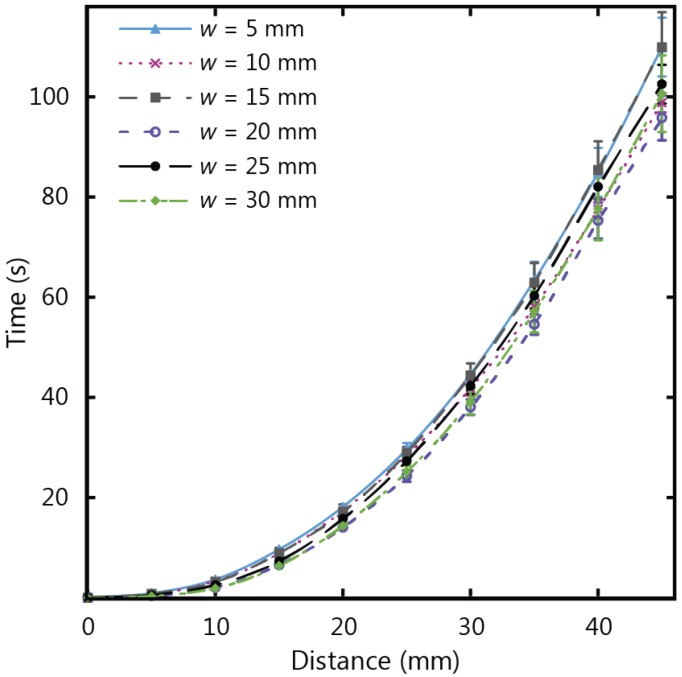
Experimental results for wicking in 17 CHR strips of widths (*w*) varying from 5 to 30 mm, and a length of 45 mm.

Figure 7 shows the experimental results for fluid flow in µPADs of varying width using Whatman 1 CHR, which has a thickness of 0.18 mm. The total wicking time for paper strips of 45 mm in length was observed to decrease as the width increased, corresponding to an increase of wicking speed for increasing width. This confirms that the width influences the fluid flow behaviour for thin strips, with diminishing dependency as the strip width increases. The same set of experiments were repeated with thicker chromatography paper (Whatman 17 CHR with a thickness of 0.70 mm) to give the results in Figure 8. For the thicker paper, no variations associated with width were observed within the experimental error. It is therefore crucial to consider the influence of width on the flow rates for thin paper strips with larger widths (greater than 5 mm) when larger µPADs are being designed in situations with an abundance of sample fluid volume.

Bohm *et al.* [41] also observed slower flow in channels with smaller widths for paper strips with hydrophobic barriers and widths of 1 to 5 mm, and found that the dependence on width diminished above 4 mm. This behaviour was attributed to the large number of pores that were “terminated” at the channel walls (dead-end pores) where the hydrophobic barrier was located, thus interrupting the flow and resulting in slower fluid flow (longer time to fill a given volume of space). Our observations were for strips with cut edges and we found the dependence to extend beyond widths of 4 mm, up to 40 mm, however the dead-end pore mechanism also provides a plausible explanation for the behaviour we observed. A video is included in the supplementary information showing side-by-side tests on a benchtop with a 10 mm strip beside a 35 mm strip. The video demonstrates the width dependence by showing the varying wicking speed for two different widths under identical conditions. The video also provides substantiation of the dead-end pore explanation since it shows that the fluid flow at the middle of the paper strip progressed quicker than the flow at the edges, indicating that the fluid near the cut edges was hindered. Since the edges are hindering the flow, wider strips would experience less hindrance than narrower strips and thus a quicker flow, which provides corroboration for the dead-end pore explanation.

### 3.6. Post-Wetting Flow

Our most unexpected observation was fluid flow in the paper strip after wetting was complete. The paper strips remained in contact with the reservoir after the liquid front had travelled the full length of the strip. Though no additional fluid transport was expected due to capillary forces, since wetting was complete, further flow, or post-wetting flow, was observed in the paper strip. Post-wetting flow caused an increase in the amount of dye in the paper strip, and was detected visually as a darkening in the colour of the paper strip. ImageJ software was used to analyze images of post-wetting flow to quantify our observations and rule out other causes such as evaporation or diffusion.

**Figure 9:**
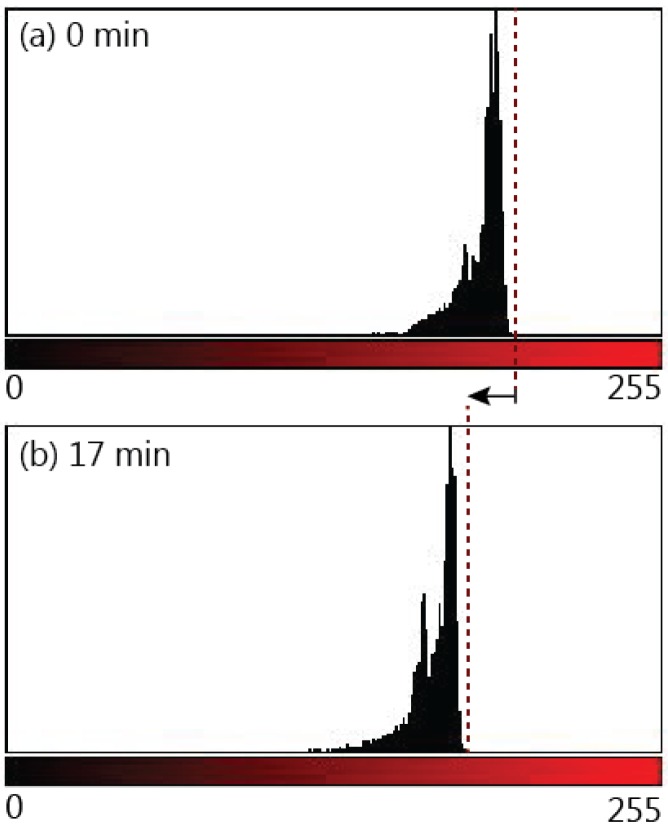
Comparison of colour profiles of the red tone in 1 CHR paper strips (**a**) upon wetting for the full length of the strip, and (**b**) 17 min after wetting to detect flow after wetting.

**Figure 10:**
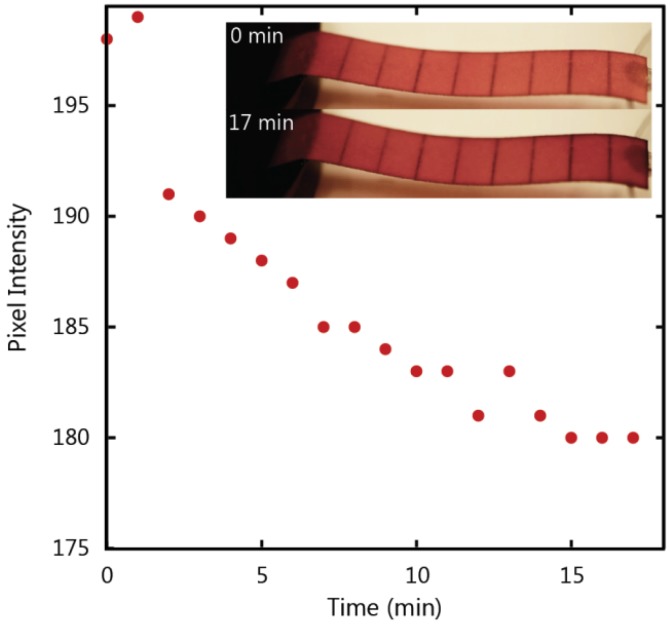
Colour intensity of a 1 CHR paper strip after wetting.

Figure 9 compares the colour intensity of a paper strip at the moment of complete wetting to the colour intensity 17 min after complete wetting. Colour intensity is measured by representing each pixel in the image with a numerical value between 0 and 255, where 0 is the value assigned to the darkest pixels and 255 is assigned to the brightest red pixels. The colour profile shows a shift to the left (lower pixel values) that reflects the darkening in colour due to the increase in dye within the paper strip. The darkening can also be observed in the paper strips in the inset of Figure 10. The overall darkening of the whole paper strip confirms that the amount of dye increased due to post-wetting flow and not due to diffusion, since diffusion would result in a redistribution of the dye rather than an overall increase. To provide further verification of whether the colour change was due to increased flow from the reservoir or the diffusion of dye particles, a fully wetted strip was removed from the reservoir immediately after the fluid front reached the end of the strip and the colour was compared to a strip that remained in the reservoir. A slight redistribution of the dye was detected as the removed strip evened in colour, however, there was no distinct darkening in the colour of this strip. Additionally, the mass of both strips was measured 18 min after wetting. Both strips were kept in the same location to ensure that both would undergo the same amount of evaporation and rule out evaporation as a cause of the change in fluid volume within the strip. The mass of the paper strip that remained in the reservoir was 36% higher than the paper strip that was removed from the reservoir, thus confirming the presence of post-wetting flow.

To determine the post-wetting flow behaviour as a function of time we analyzed the colour intensity of the paper strip after wetting was complete. The maximum value for red pixels is the numerical value assigned by the software to the brightest red pixel in the image. This value was measured every minute after saturation, where a lower value represents a darker red colour corresponding to an increase in dye concentration, and these values are plotted in Figure 10. The data demonstrates a decreasing relationship between time and brightness of the red colour in the paper strip that levels off after approximately 15 min. This data indicates that there exists a maximum quantity of water that will fill the paper strip during post-wetting flow.

**Figure 11:**
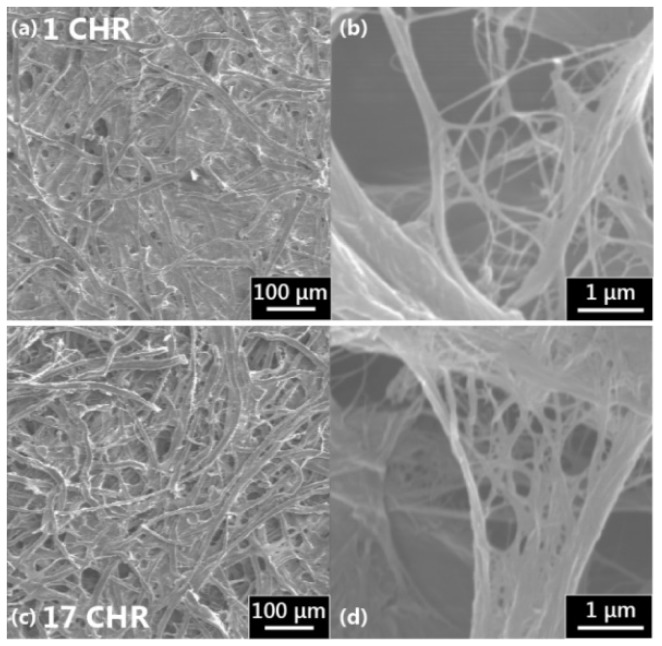
Scanning electron micrographs of cellulose fibre networks in (**a**) 1 CHR at 100× magnification, (**b**) 1 CHR at 10,000×, (**c**) 17 CHR at 100×, and (**d**) 17 CHR at 10,000×.

A close look at the fibre structure of paper, as seen in the SEM micrograph in Figure 11, shows that interfibre pores are created by the spaces between fibres, which tend to be highly variable in size [38,42]. A study by Roberts *et al.* observed that the bulk filling of pores is not the primary flow mechanism in paper, rather it is flow along the channels caused by fibre overlap, in other words, capillary driven film flow. The cellulose fibres also contain pores within the fibre, known as intrafibre pores [37,38]. Filling of these intrafibre pores leads to fibre swelling, while the capillary forces of a liquid between fibres can cause fibre deformation [37,43,44]. While these fibre swelling effects are not immediately apparent during imbibition, they could provide an explanation for the presence of post-wetting flow. Movement of the fibres due to stretching or relaxation caused by elastic forces could also account for fluid flow within the paper after wetting.

For design of precise µPADs when using a large fluid reservoir, it will be important to consider the post-wetting effects in relation to the intensity of colorimetric signals by controlling the time of submersion and elapsed time prior to test readout by the user. This phenomenon is of particular interest for paper-based microfluidic applications where high sample volumes are readily available.

## 4. Conclusions

In this paper, we investigated the impact of surrounding conditions such as temperature and humidity, paper machine direction, strip length, and strip width on fluid flow behaviour in paper strips fed from a large fluid reservoir. Using experimental data, we determined that length and humidity do not have a significant impact on flow behaviour in paper strips for the conditions we tested. We noticed quicker wicking for flow parallel to the machine direction of the paper compared to the cross (perpendicular) direction. Our experimental results indicated that the wicking time decreases as temperature increases in proportion to decreases in the inverse root of the fluid viscosity. Additionally, we observed that wicking time also decreases with increasing width for thin (1 CHR) paper strips, a dependency which diminishes as width increases. Width was not found to influence the wicking behaviour for the thicker paper strips (17 CHR). We also observed post-wetting flow when the paper strips remained in contact with a fluid reservoir, which should be considered during design of µPADs used in conjunction with large sample volumes, for example, in environmental testing applications.

## Figures and Tables

**Table 1:** Properties of Whatman chromatography paper.

Paper Type	Basis Weight (g/m^2^)	Thickness (mm)	Porosity	Flow Rate (mm/min)
Whatman 1 CHR	87	0.18	67.8%	4.33
Whatman 17 CHR	325	0.70	69.1%	6.33

## Supplementary Materials

The following are available online at http://www.mdpi.com/2072-666X/7/5/73/s1, Video S1: Side-by-side tests on a benchtop with a 10 mm strip beside a 35 mm strip.
